# A novel formamidase is required for riboflavin biosynthesis in invasive bacteria

**DOI:** 10.1016/j.jbc.2022.102377

**Published:** 2022-08-13

**Authors:** Svetlana N. Yurgel, Skylar A. Johnson, Jennifer Rice, Na Sa, Clayton Bailes, John Baumgartner, Josh E. Pitzer, R. Martin Roop, Sanja Roje

**Affiliations:** 1 Grain Legume Genetics and Physiology Research Unit, USDA, ARS, Prosser, Washington, USA; 2 Institute of Biological Chemistry, Washington State University, Pullman, Washington, USA; 3 Department of Microbiology and Immunology, Brody School of Medicine, East Carolina University, Greenville, North Carolina, USA

**Keywords:** riboflavin biosynthesis, invasive bacteria, formamidase, flavins, AFRPP, 2-amino-5-formylamino-6-ribosylaminopyrimidin-4(3H)-one 5′-monophosphate, DARoPP, 2,5-diamino-6-ribosylamino-4(3H)-pyrimidinone 5′-phosphate, GCHIII, GTP cyclohydrolase III, LB, Luria–Bertani, MjGC, *Methanocaldococcus jannaschii* GTP cyclohydrolase II, MMNH_4_, Min-mannitol-NH_4_+, RBP, RF biosynthesis pathway, RF, riboflavin, SBA, Schaedler blood agar, Sm-BrbF, *Sinorhizobium meliloti* BrbF

## Abstract

Biosynthesis of riboflavin (RF), the precursor of the redox cofactors FMN and FAD, was thought to be well understood in bacteria, with all the pathway enzymes presumed to be known and essential. Our previous research has challenged this view by showing that, in the bacterium *Sinorhizobium meliloti*, deletion of the *ribBA* gene encoding the enzyme that catalyzes the initial steps on the RF biosynthesis pathway only causes a reduction in flavin secretion rather than RF auxotrophy. This finding led us to hypothesize that RibBA participates in the biosynthesis of flavins destined for secretion, whereas *S. meliloti* has another enzyme that performs this function for internal cellular metabolism. Here, we identify and biochemically characterize a novel formamidase (SMc02977) involved in the production of RF for intracellular functions in *S. meliloti*. This catalyst, which we named Sm-BrbF, releases formate from the early RF precursor 2-amino-5-formylamino-6-ribosylamino-4(3H)-pyrimidinone 5′-phosphate to yield 2,5-diamino-6-ribosylamino-4(3H)-pyrimidinone 5′-phosphate. We show that homologs of this enzyme are present in many bacteria, are highly abundant in the Rhizobiales order, and that sequence homologs from *Brucella abortus* and *Liberobacter solanacearum* complement the RF auxotrophy of the Sm1021ΔSMc02977 mutant. Furthermore, we show that the *B. abortus* enzyme (Bab2_0247, Ba-BrbF) is also an 2-amino-5-formylamino-6-ribosylamino-4(3H)-pyrimidinone 5′-phosphate formamidase, and that the *bab2_0247* mutant is a RF auxotroph exhibiting a lower level of intracellular infection than the wildtype strain. Finally, we show that Sm-BrbF and Ba-BrbF directly interact with other RF biosynthesis pathway enzymes. Together, our results provide novel insight into the intricacies of RF biosynthesis in bacteria.

Organic cofactors are required for activity of many enzymes that carry out the cell’s biochemical reactions. These cofactors or their direct precursors are classified as vitamins for organisms that need to obtain them from food because they are essential for the survival. The redox cofactors FMN and FAD and their precursor riboflavin (RF) are the three vitamers of vitamin B_2_. FMN and FAD play important roles in many cellular processes, including respiration, DNA repair, biosyntheses of heme groups, cofactors, and nucleotides, fatty acid beta-oxidation, bioluminescence, and light sensing (1). In contrast to animals, which are unable to synthesize RF but are efficient at importing it from the environment, bacteria rely on biosynthesis to satisfy their need for flavins. Consequently, blocking RF biosynthesis can inhibit prototrophic pathogens but not their animal hosts. Therefore, the inhibitors of RF synthase and lumazine synthase, catalyzing the last two steps on the RF biosynthesis pathway (RBP), have been explored as the targets for design of new antibiotics (1, 2).

The current view of RBP assumes that all reactions and enzymes of RF biosynthesis in bacteria are known and essential. The RBP in bacteria (Fig. 1) starts with the reaction catalyzed by GTP cyclohydrolase II enzymes (GTPHII, RibA), which proceeds in two steps. The first step consists of the ring cleavage and dephosphorylation on GTP, producing 2-amino-5-formylamino-6-ribosylaminopyrimidin-4(3H)-one 5′-monophosphate (AFRPP) as the reaction intermediate. The second step is deformylation of this intermediate to produce 2,5-diamino-6-ribosylamino-4(3H)-pyrimidinone 5′-phosphate (DARoPP) and formate. In Archaea, these two steps are catalyzed by two different archaea-specific enzymes: GTP cyclohydrolase III (GCHIII) produces the formylamino-pyrimidine nucleotide intermediate, whereas the formamide hydrolase (ArfB) deformylates this intermediate into DARoPP (3, 4) (Fig. 1). The remaining steps are catalyzed by the dihydroxy-butanone phosphate synthase (RibB), bifunctional deaminase-reductase (RibD), lumazine synthase (RibH), and RF synthase (RibE) in bacteria and Archaea (1, 5).

**Figure 1:**
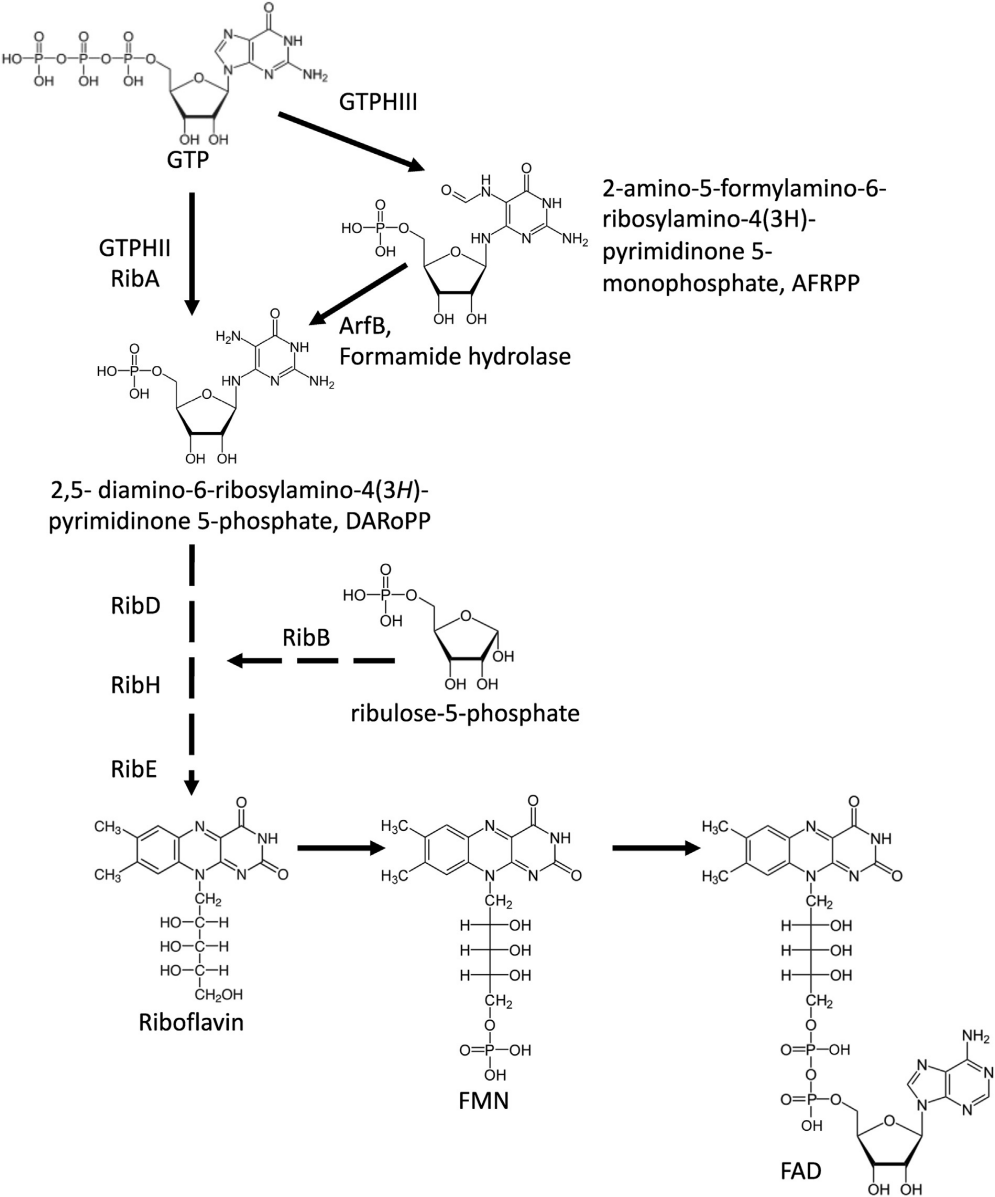
**RF biosynthesis in bacteria and archaea.** GTPHIII, GTP cyclohydrolase III; GTPHII, GTP cyclohydrolase ll; RF, riboflavin.

*Sinorhizobium meliloti* belongs to the Rhizobiales order of α-proteobacteria that also includes animal and human pathogens like *Brucella* and *Bartonella*. Similar to pathogenic bacteria, which invade host cells and modify the host intracellular environment (6), *S. meliloti* invades root cells of the host plants and triggers development of symbiotic organs—root nodules where the bacteria fix atmospheric nitrogen and provide it to the host plant for growth (7). All known proteins comprising the RBP in bacteria were found in the *S. meliloti* Rm1021 genome (8).

In *S. meliloti*, the *ribBA* gene codes for a putative bifunctional enzyme with dihydroxy-butanone phosphate synthase (*ribB* domain) and GTP cyclohydrolase II (*ribA* domain) activities, catalyzing the initial steps of the RBP. We have previously shown that an in-frame deletion of the full-length *ribBA* does not cause RF auxotrophy in *S. meliloti*, suggesting that there are other enzyme(s) that can catalyze this reaction and prevent RF starvation in the absence of RibBA (9). On the other hand, the loss of RibBA affected the ability of *S. meliloti* to secrete flavins. Based on these results, we hypothesize that *S. meliloti* has two partly interchangeable modules for biosynthesis of RF: one fulfilling the internal need for flavins in the bacterial metabolism, and the other producing RF for secretion and involving the RibBA (9). Likewise, a recent study proposed a modular structure of bacterial RBP to supply RF for specific intracellular and extracellular functions (10). In this study, we identified and biochemically characterized the novel *S. meliloti* enzyme involved in production of RF for intracellular functions. We also showed that homologs of this protein are present in many bacteria and some Archaeal phyla and are highly abundant in the Rhizobiales order. Our findings show that the strategies used by various organisms to fulfill their needs for the essential cofactors FMN and FAD are more varied than previously thought and point to the bacterial alternative to ArfB enzymes (which we named BrbF for bacterial riboflavin biosynthesis formamidase) as a potential target for combating a class of pathogenic bacteria.

## Results

### Identification of a novel enzyme required for the RF biosynthesis in α-proteobacteria

**Figure 2:**
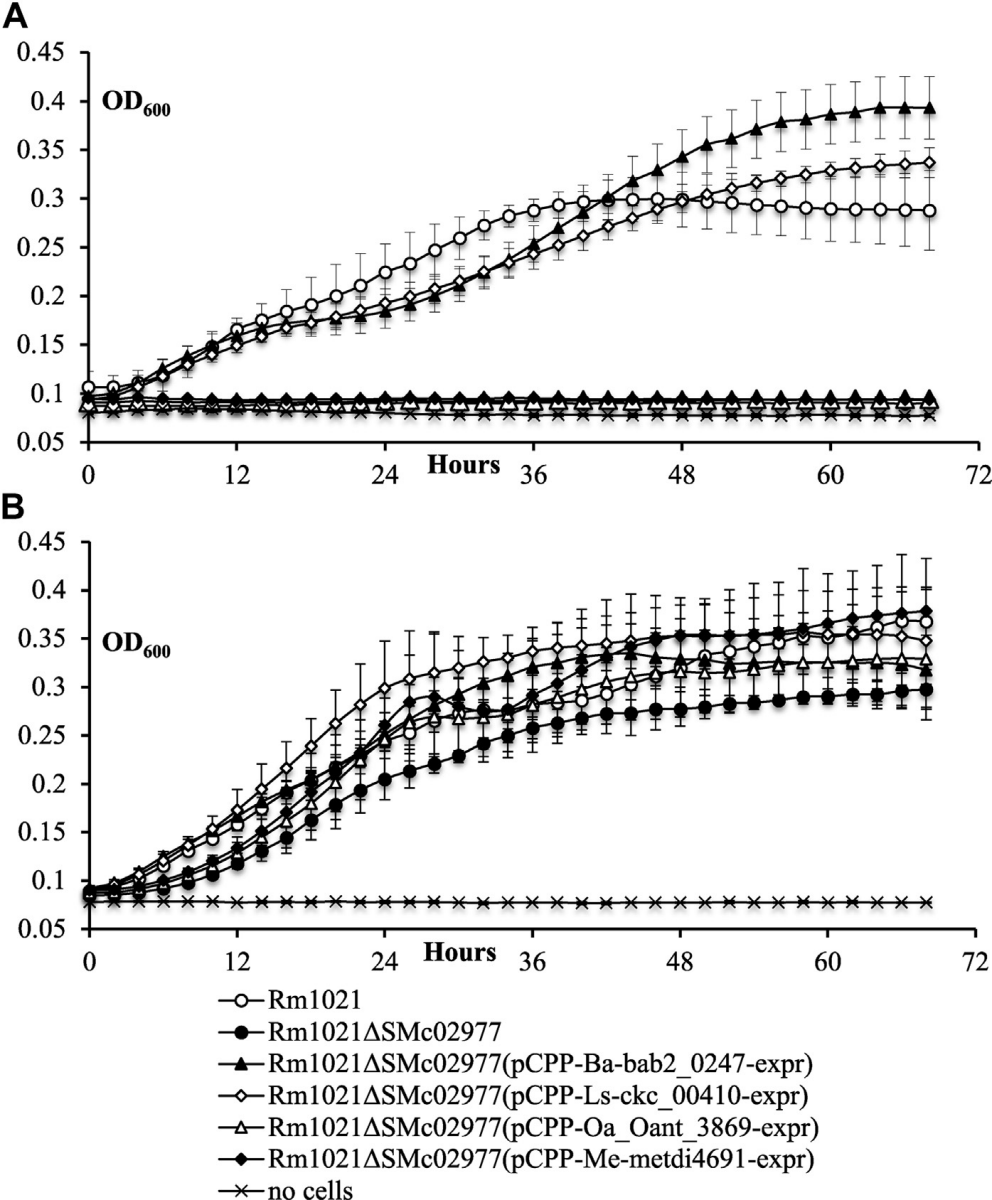
**Growth of the *Sinorhizobium meliloti rib* mutants.***S. meliloti* strains were grown in (*A*) Min-mannitol-NH_4_ (MMNH_4_) media or in (*B*) MMNH_4_ media supplemented with 250 μM riboflavin. Data are the average ± SD. Three biological replicates were used for each measurement.

Our previous findings showed that the RibBA enzyme, which catalyzes the initial steps on the RBP in bacteria (Fig. 1), is essential for flavin secretion but not for survival in *S. meliloti* (9). This led us to hypothesize that another enzyme is required for catalysis of the initial step on the RBP that produces flavins for the internal metabolic needs of these bacteria. To identify that enzyme, close to 6000 mini-Tn*5*-mutagenized *S. meliloti* Rm1021Δ*ribBA* colonies were screened for the ability to grow without exogenous RF. Several RF auxotroph (RF^–^) mutants were identified in the screen. The DNA sequences adjacent to the inserted Tn*5* transposon were in selected mutants identified using the arbitrary PCR method (11). In two mutants, Rm1021Δ*ribBA*-RFA-12 and Rm1021Δ*ribBA*-RFA-Σ (Table S1), the transposon was located in the ORF SMc02977, which has been annotated as a putative creatinine amidohydrolase (8), an enzyme catalyzing the reversible conversion of creatinine to creatine. Using phage μ12-mediated transduction (12), we verified that the RF^–^ phenotype of Rm1021Δ*ribBA*-RFA-12 and Rm1021Δ*ribBA*-RFA-Σ was caused by the Tn*5* insertion, since all Rm1021Δ*ribBA* transductants that carried neomycin resistance (Nm^r^) from original mutants into 1021Δ*ribBA* also required RF for growth. When Nm^r^ markers from Rm1021Δ*ribBA*-RFA-12 and Rm1021Δ*ribBA*-RFA-Σ were transduced into the wildtype strain Rm1021, the resulting transductants also exhibited the RF^–^ phenotype (Table S1). Next, we introduced in-frame deletions of the entire SMc02977 in Rm1021 genome using the recombination-replacement strategy (13). Unlike *ribBA* deletion (9), SMc02977 deletion prevented *S. meliloti* from growing without the exogenous RF (Fig. 2 and Table S1), indicating that SMc02977 was essential for RF biosynthesis in *S. meliloti*. The strain 1021ΔSMc02977 could not nodulate alfalfa (data not shown) most probably because of its inability to survive and function without a high concentration of RF in the environment.

### SMc02977 deformylates AFRPP to DARoPP

The SMc02977 protein was produced as a recombinant protein in *Escherichia coli* and purified to determine its biochemical function. For purification of the recombinant protein, the ORF of *S. meliloti* SMc02977 was ligated into the pET-30 Ek/LIC vector, the resulting plasmid was transformed into the BL21 strain of *E. coli*, and the recombinant protein was affinity purified from the cultures induced with IPTG. Initial enzyme assays of the purified protein showed that it was unable to hydrolyze GTP (not shown), which led us to hypothesize that SMc02977 catalyzes only the deformylation of AFRPP to produce DARoPP and formate, and that the ring cleavage and dephosphorylation on GTP are catalyzed by another, yet to be discovered enzyme. To test this hypothesis, the ArfA enzyme from *Methanococcus janaschii* was produced as a recombinant protein in *E. coli*, purified, and used to synthesize AFRPP from GTP. Activity assays with the purified SMc02977 and AFRPP as a substrate determined that the SMc02977 protein releases formate from AFRPP to yield DARoPP (Fig. S1). Thus, this protein has the same catalytic function as the archaeal ArfB enzyme and was therefore renamed to Sm-BrbF (*S. meliloti* BrbF; for bacterial riboflavin biosynthesis formamidase). Its catalytic properties, as calculated using the SigmaPlot Enzyme Kinetics Module, revealed that Sm-BrbF operates with standard Michaelis–Menten kinetics, with a *k*_*m*_ = 152.44 ± 33.59 μM, and *V*_max_ = 546.26 ± 40.31 nmol/min/mg. Activity assays based on fluorescence-HPLC detection of derivatized DARoPP or formate at 0.5 mM AFRPP concentration produced similar results: 290 ± 11 nmol/min/mg and 272 ± 42 nmol/min/mg, respectively.

Providing another line of evidence in support of the Sm-BrbF enzyme catalyzing deformylation of AFRPP to DARoPP, the DARoPP content of the wildtype *S. meliloti* 1021 strain was small but detectable at 1.76 ± 0.3 pmol/mg of fresh bacterial cells, whereas that of the 1021ΔSMc02977 bacteria was below the detection limit of the assay (<20% of the wildtype level).

### Similarity searches found that sequence homologs of Sm-BafB are widespread among bacteria

To find out if Sm-BrbF homologs are restricted to a narrow phylogenetic group of bacteria, or relatively widespread, we conducted a comprehensive similarity search for the sequence homologs of the full-length protein. The Sm-BrbF homologs were found in many bacterial taxa and in some Archaea (Fig. S2). However, they were most frequently found in alpha-proteobacteria, especially in the *Rhizobium*-*Agrobacterium*, *Sinorhizobium*, *Bartonella*, and *Brucellaceae* groups. Furthermore, in the *Rhizobium-Agrobacterium*, *Sinorhizobium*, and *Brucellaceae* groups, the genes encoding Sm-BrbF homologs were located in conserved genetic regions containing gene homologs of the putative transcriptional regulator MarR (SMc02976) and the putative cobalamin biosynthetic protein CobW (SMc2978). To investigate the role of these proteins in RF biosynthesis, we introduced in-frame deletions of the entire SMc02976 and SMc29878 ORFs in Rm1021 genome using recombination-replacement strategy. The deletion of neither of the genes resulted in RF auxotrophy (Fig. S3), indicating that the proteins encoded by SMc02976 and SMc29878 do not play an essential role in intracellular RF production in Rm1021.

Based on sequence homology to the enzyme catalyzing the hydrolysis of creatinine to creatine in *Pseudomonas putida* (14), Sm-BrbF was initially annotated as putative creatinine amidohydrolase/creatininase (8). Currently, 708 creatininases are annotated in the Kyoto Encyclopedia of Genes and Genomes database. Mechanistically, creatininases are hydrolases that act on carbon–nitrogen bonds other than peptide bonds, specifically on cyclic amides. The classical GTP cyclohydrolase II enzymes also act on a cyclic amide, converting GTP to DARoPP, formate, and pyrophosphate (15, 16) (Fig. S4). To determine the phylogenetic relationship between Sm-BrbF, the *P. putida* creatininase, and the *S. meliloti* GTP cyclohydrolase II domain (RibA), the sequences of several distinct proteins from creatininase and RibA groups were also included into the analysis together with representative Sm-BrbF homologs.

**Figure 3:**
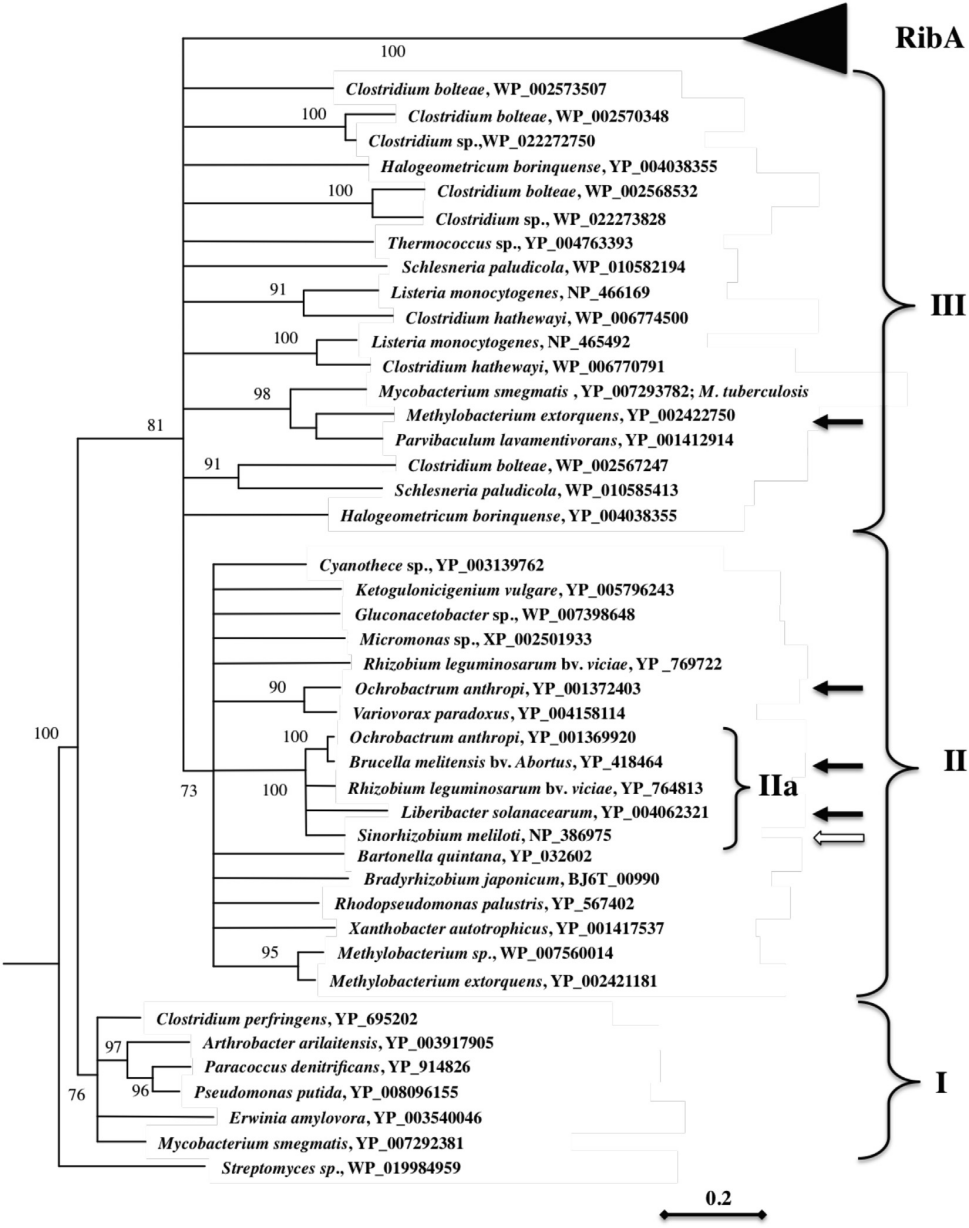
**Maximum-likelihood distance tree based on sequence alignment of SMc02977 sequence (bootstrap value >700).** Bootstrap values (as percentage) are shown to indicate the level of support for the different parts of the tree topology shown. *Black triangle* indicates the position of the GCHII/RibA clade. *White arrow* indicates the position of SMc02977. *Black arrows* indicate the proteins used in the complementation analysis. GCHII, GCHIII, GTP cyclohydrolase II.

The maximum-likelihood distance tree based on these protein sequences is shown in Figure 3. In this tree, the homologs of full-length Sm-BrbF form distinct clusters, which are separated from the GTP cyclohydrolase II domain of RibA proteins by a long branch (bootstrap value = 1000). The SMc02977 homologs form three well-supported clades (bootstrap value = >700, Fig. 2): (i) clade I, containing close homologs of the *P. putida* creatininase; (ii) clade II, containing close homologs of the *S. meliloti* SMc02977; and (iii) clade III, containing a number of uncharacterized proteins from a wide spectrum of bacteria, including human pathogens *Clostridium* spp, *Mycobacterium* spp, and *Listeria monocytogenes*. The Rhizobiaceae family of α-proteobacteria forms a strongly supported subcluster (Fig. 3IIa). In addition, in a number of invasive bacteria such as *Clostridium* spp, *L. monocytogenes*, *Ochrobactrum anthropi*, and *Rhizobium leguminosarum*, two distinct Sm-BafB homologs were identified, whereas others, such as *Brucella abortus*, *Sinorhizobium melioti*, and *Mycobacterium tuberculosis* possess a single sequence homolog of Sm-BafB.

### The Sm-BafB homologs from *B. abortus* and *Liberobacter solanacearum* complement RF auxotrophy of Sm1021ΔSMc02977

To find out if selected proteins that are phylogenetically related to Sm-BrbF perform the same catalytic function, the homologs from *B. abortus* 2308 (Bab2_0247) and *L. solanacearum* CLso-ZC1 (Ckc_00410) were overexpressed in Rm1021ΔSMc02977 from a broad host range plasmid pCPP30 (17) under native promoters. The expression of both proteins restored the ability of the strain to grow without exogenous RF (Fig. 2 and Table S1), indicating functional equivalency between these proteins and SMc02977. On the other hand, when the more distant Sm-BrbF homologs from *O. anthropi* American Type Culture Collection 49188 (Oant_3869) (cluster II) and *Methylobacterium extorquens* DM4 (Metdi4691) (cluster III) were overexpressed in Rm1021ΔSMc02977 under the *S. meliloti* Rm1021 SMc02977 promoter, the resulting strains still required RF supplementation for growth (Fig. 2 and Table S1).

### The Sm-BrbF homolog Bab2_0247 plays an essential role in RF biosynthesis in the *B. abortus* 2308 strain

*B. abortus* is a dangerous animal pathogen, and the understanding of the role of the alternative branch of the RF biosynthetic pathway in *Brucella* infection is a fundamental question in bacteria–animal pathogenesis. We constructed in-frame deletions of *bab2_0247* and *bab1_0455* (3,4-dihydroxy-2-butanone 4-phosphate synthase:GTP cyclohydrolase II) in the *B. abortus* 2308 genome. The *B. abortus* mutant missing *bab2_0247*, JB32, required RF supplementation for growth and exhibited a lower level of intracellular infection than the wildtype strain (Figs. S5 and S6), pointing to the essential role of Bab2_0247 in the intracellular RF production. On the other hand, similarly to the *S. meliloti* mutant (9), *B. abortus* 2308Δ*ribBA*, JB24, was an RF prototroph (Fig. S5) and capable of establishing intracellular infection in C57BL/6^Nramp1+/+^ mice at the rate comparable to the wildtype strain (Fig. S6), indicating dispensability of *ribBA* in the *B. abortus* intracellular RF metabolism and pathogenesis.

In order to test for enzyme activity, the ORF of Bab2_0247 was ligated into the pET-30 Ek/LIC vector, the resulting plasmid was transformed into the BL21 strain of *E. coli*, and the recombinant protein was affinity purified from the cultures induced with IPTG, and assayed as described for Sm-BrbF. The activity assays showed that Bab2_0247 indeed hydrolyzes AFRPP to DARoPP with V = 467.4 ± 128.3 nmol min^−1^ mg^−1^ at 1 mM AFRPP substrate concentration.

### The *S. meliloti* Sm-BrbF and *B. abortus* Bab2_0247 form complexes with other enzymes on the RF biosynthetic pathway

**Figure 4:**
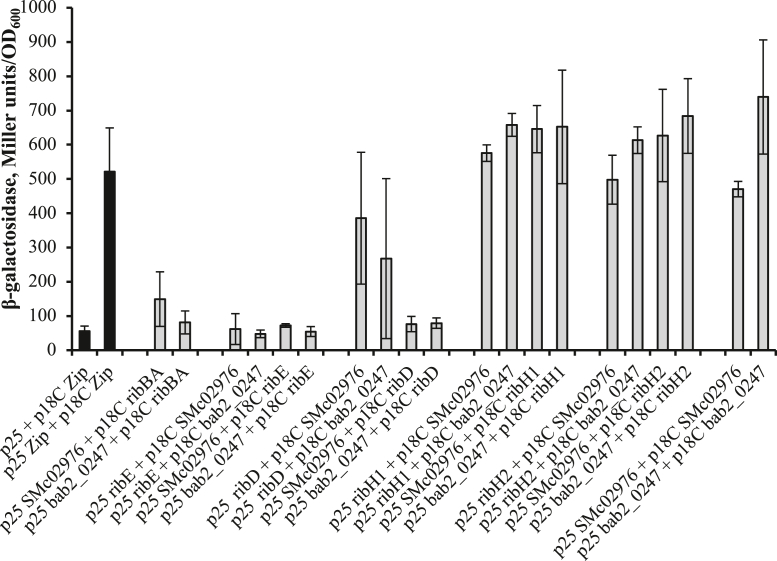
**Bacterial two-hybrid analysis of interaction between enzymes of the *Sinorhizobium meliloti* RBP and *S. meliloti* SMc02977 and *Brucella abortus* bab2_0247 proteins.** Interaction between Rib proteins is shown in *gray*, and controls are shown in *black*. RibH1/SMc01777 and RibH2/SMc01609, 6,7-dimethyl-8-ribityllumazine synthase; RibE/SMc0177, riboflavin synthase; RibBA/SMc00008, bifunctional dihydroxybutanone phosphate synthase and GTP cyclohydrolase II. Data are the average ± SD. Three biological replicates were used for each measurement.

We used the bacterial adenylate cyclase two-hybrid system (18) to test the ability of Sm-BrbF and Bab2_0247 to form complexes with other *S. meliloti rib* proteins. We did not detect any significant interaction of Sm-BrbF or Bab2_0247 with RibBA and RibE proteins. The adenylate cyclase activity in the cells carrying Sm-BrbF or Bab2_0247 and RibD proteins fused to adenylate cyclase domains was inconsistent (Fig. 4). On the other hand, strong interaction of Sm-BrbF and Bab2_0247 with both RibH1 and RibH2 was detected. In addition, Sm-BrbF and Bab2_0247 can interact with themselves producing strong adenylate cyclase activity suggesting a multiunit structure of Sm-BrbF and Bab2_0247 enzymes *in vivo*.

## Discussion

The finding that the deletion of the bifunctional dihydroxy-butanone phosphate synthase:GTP cyclohydrolase II, RibBA, in *S. meliloti* did not result in RF auxotrophy but decreased bacterial flavin secretion (9) suggested a higher complexity of the RF biosynthetic pathway in *S. meliloti* and potentially in other bacteria, than previously thought. More specifically, it implied a potential modularization of RBP in bacteria and an existence of alternative enzymes compensating for the loss of RibBA. As a result of this study, a novel protein, encoded by SMc02977 and essential for RF biosynthesis in *S. meliloti*, was identified.

SMc02977 was initially annotated as a putative creatinine amidohydrolase acting on carbon–nitrogen bonds other than peptide bonds, specifically in cyclic amides (8). However, our data indicated that this newly identified protein acts as a formamide hydrolase, catalyzing deformylation of AFRPP into DARoPP. Thus, despite the lack of sequence similarity, this novel enzyme is catalyzing the same reaction as the ArfB protein of the archaeal RBP (3, 4) and was therefore named BrbF. In order to completely replace the catalytic functions of the RibBA enzyme, yet to be discovered in BrbF-containing bacteria are the GCHIII, catalyzing the biosynthesis of AFRPP from GTP, and the alternative dihydroxy-butanone phosphate synthase (RibB) (Fig. 1). Our bioinformatic analysis neither did identify any *S. meliloti* proteins with homology to the archaeal GCHIII nor did it find a putative second RibB. Moreover, our Tn5 mutagenesis did not identify any novel enzymes potentially involved in RBP in addition to the SmBrbF reported here. Therefore, additional work is needed to identify these missing catalysts.

The Sm-BrbF sequence homologs were found in a number of bacteria from the *Rhizobium*-*Agrobacterium*, *Sinorhizobium*, *Bartonella*, and *Brucellaceae* groups. *B. abortus and L. solanacearum* BrbF homologs complemented the RF auxotrophy of Rm1021ΔSMc02977, pointing to the functional similarities between these proteins. Furthermore, deletion of Sm-BrbF homolog (Bab2_0247) in *B. abortus* 2308 induced RF deficiency in the mutant. These data indicated that the Sm-BrbF homologs might also be essential for RF biosynthesis in other bacteria.

The question of the reactions catalyzed by the more distant sequence homologs of the Sm-BrbF, belonging to clades II and III, remains open. In addition, in a number of pathogenic bacteria such as *Clostridium* spp, *L. monocytogenes*, and *O. anthropi*, two distinct proteins related to Sm-BrbF were annotated. Interestingly, the more distant *O. anthropi* BrbF homolog, Oant_3869, did not complement Rm1021ΔSMc02977. It is possible that Oant_3869 does not possess formamide hydrolase activity in general, or that it cannot function in the *S. meliloti* background, for example, because of inability to form a complex with the *S. meliloti* RibH proteins.

It has been hypothesized that intragenome conserved sequence homologs provide bacteria with adaptive benefits likely because of increased regulatory flexibility and/or increased gene dosage (19). Similarly, the presence of redundant branches of RBP may channel the critical pathway intermediates toward interaction with different downstream cytoplasmic or membrane RBP proteins, therefore defining localization of the final flavin products. Flavin secretion likely plays a significant role in the interaction of bacteria with eukaryotic host cells (10), providing advantage for establishment of bacterial colonization of the host surface and internal tissue. Bacterial flavins were shown to be important in *Brucella* virulence (20, 21) and the *S. meliloti* host–plant root colonization and nodulation competitiveness (9, 22, 23). An RF breakdown product, lumichrome, affects plant root respiration, net carbon assimilation, and net biomass in several plant species (22, 24, 25, 26), pointing to the potential of bacteria-derived flavins to act as plant growth–promoting molecules. The data presented in this study support our hypothesis that two modules for RF biosynthesis exist in *S. meliloti*, one of them using the bifunctional RibBA to produce flavins targeted for secretion and the other using the alternative enzymes, including the BrbF characterized in this study, to produce RF for the internal needs of the bacteria (9). In addition, the presence of Sm-BrbF homologs in many taxa of alpha-proteobacteria that contain multiple invasive species suggests a possibility for the universal importance of flavin secretion in bacteria–host interactions and pathogen invasion.

Based on the data presented here, we hypothesize that Sm-BrbF is, with yet to be discovered GCHIII (BrbG for bacterial riboflavin biosynthesis GTP cyclohydrolase III) and an alternative dihydroxy-butanone phosphate synthase (RibB2), a part of the metabolon involved in the intracellular flavin metabolism, whereas a separate RibBA-containing metabolon produces flavins for secretion. We speculate that the latter metabolon may be bound to the cellular membrane, forming a complex with a putative transporter that secretes flavins outside the bacteria immediately following the production. We expect that the choice of whether DARoPP (the product of BrbG + BrbF or RibA) and dihydroxy-butanone phosphate (the product of RibB) will be used internally or secreted outside the cell is made at the level of RibBA *versus* BrbG:BrbF:RibB2 branches and is maintained till the last step of RBP. This implies that either both branches are part of two different metabolons, which utilize the same well-known enzymes comprising the downstream part of RBP (Fig. 1), or that there are other unknown enzymes involved in RF biosynthesis. The use of bacterial two-hybrid system showed an interaction of lumazine synthases RibH1 and RibH2 with RibBA (27), and the data from this study showed strong interaction between RibH1 and RibH2 and BrbF, indicating that both RibBA and BrbG:BrbF:RibB2 branches could utilize at least some known RBP enzymes. Nevertheless, a deeper analysis of RBP is necessary to fully understand the complexities of RF metabolism in bacteria.

## Experimental procedures

### Bacterial strains, plasmids, and media

The bacterial strains and plasmids used in the study are listed in Table S2. The *S. meliloti* strains were grown at 30 °C on Luria–Bertani (LB) (28), MMNH_4_ (Min-mannitol-NH_4_^+^), or YMB medium (29). The *E. coli* strains were grown at 37 °C on LB medium (28). The antibiotics for *S. meliloti* were added at 200 μg/ml (streptomycin), 200 μg/ml (neomycin), or 10 μg/ml (tetracycline). For *E. coli*, the antibiotics were added at 10 μg/ml (tetracycline) or 40 μg/ml (kanamycin). Sigma–Aldrich agar was used for media preparation. All genetic manipulations with the *S. meliloti* that involve SMc02977 region were done on plates supplemented with 250 μM RF. Strains not requiring RF were routinely grown on media without RF.

### Genetic techniques

DNA manipulations were carried out following standard procedures (30). Transposon transduction and arbitrary PCR was done as described previously (11, 12).

### Transposon mutagenesis

*S. meliloti* Rm1021Δ*ribBA* was mutagenized with Tn*5*-110 using suicide plasmid pJG110 (31). After overnight mating of Rm1021Δ*ribBA* with *E. coli* S17-1 (pJG110), transposon insertion mutants were selected on MM-NH_4_ neomycin medium supplemented with RF. The individual neomycin–streptomycin resistant *S. meliloti* colonies were replicated onto both MMNH_4_ + RF and plain MMNH_4_ medium. Colonies that are unable to grow on the plain MMNH_4_ medium were picked and further purified to verify RF auxotrophy. Transposon insertions were located using an arbitrary PCR (11, 32) to determine DNA sequences adjacent to the Tn*5*.

### Construction of in-frame deletion mutants in *S. meliloti*

The SMc02977, SMc02976, and SMc02978 deletion mutants in Rm1021 were constructed using an insertion/excision strategy (13) with some modifications. Sucrose selection of the mutants was done on YMB plates supplemented with 5% sucrose and 0.5 mM RF. To construct the *S. meliloti* deletion mutants, PCR was used to amplify the two chromosomal regions (0.5 kb and 0.6 kb) flanking the regions to be deleted, using primers listed in Table S3. The deletions were confirmed by PCR using primers located outside the regions flanking those used to construct the deletions. The ability of the strains to grow without RF was tested after verification of the gene excision.

### Construction of in-frame deletion mutants in *B. abortus*

The Bab2_0247 and Bab1_0455 deletion mutants in Ba 2308 were constructed using an insertion/excision strategy. After the upstream and downstream regions are amplified using primers listed in Table S3, the PCR products are gel purified and then used in a single PCR. The 2 kb PCR products were gel extracted and cloned into the SmaI site of *sacB*-based counterselection *K*_*m*_-resistant plasmid pNPTS193 (33). The resulting plasmids were called pNPTSΔ0247 and pNPTSΔ00455. The constructs were confirmed by sequencing to be a nonpolar unmarked deletion of *bab2_0247* and *bab1_0455*. pNPTSΔ0247 and pNPTSΔ0455 were electroporated into a freshly struck culture of Ba 2308, recovered in *Brucella* broth (BD) for 1 h shaking at 37 °C, and plated on Schaedler blood agar (SBA; BD) containing 5% defibrinated bovine blood (Quad Five) and 45 μg/ml Km to select for single crossovers. Plates were incubated at 37 °C in the presence of 5% CO_2_ for at least 48 h until colonies formed. One colony was picked into 1 ml BD and incubated for 4 to 6 h shaking at 37 °C in the presence of 5% CO_2_ and 0.5 mM RF. This allows for the second crossover event to occur deleting the gene. About 10 μl of the culture were spread on SBA supplemented with 10% sucrose and 0.5 mM RF and incubated for 48 h at 37 °C in the presence of 5% CO_2_. Colonies were replica plated onto SBA containing 10% sucrose and 0.5 mM RF and SBA *K*_*m*_ (45 μg/μl) and incubated 48 h at 37 °C in the presence of 5% CO_2_. Colony PCR was done on sucrose-resistant/*K*_*m*_-sensitive colonies using BAB2_0247 and BAB1_0455 up forward and down reverse primers, respectively, and wildtype Ba 2308 for control. Bands of the correct size were gel extracted and sent off for sequencing for confirmation. Sequencing results confirmed nonpolar deletions of Bab2_0247 and Bab1_0455. The strains were named JB32 and JB24.

### Experimental infection of mice

The virulence of the *B. abortus* strains in mice was evaluated using the methods described (34). Briefly, bacterial strains were grown for 3 days on Schaedler agar supplemented with 5% defibrinated bovine blood (SBA) under 5% CO_2_, and the bacterial cells were harvested in PBS. Individual mice were inoculated with 5_104 brucellae in 100 _l PBS *via* the intraperitoneal route, and at 2 and 5 weeks postinfection, the mice were euthanized by isoflurane overdose. The number of brucellae present in the spleens of mice was determined by homogenizing these organs and serial dilution and plating of the homogenates on SBA.

Female C57BL/6 mice were purchased from Jackson Laboratories and infected at 4 weeks of age. C57BL/6^Nramp1+/+^ mice were obtained as breeding pairs from Renee Tsolis at the University of California at Davis. Both sexes of C57BL/6^Nramp1+/+^ mice ranging in age from 4 to 9 weeks were used for the experimental infections and obtained from a breed colony maintained at East Carolina University. The genotype of the C57BL/6^Nramp1+/+^ mice was confirmed by PCR analysis of DNA obtained from tail snips (35). The animals use protocols under which these experiments were performed, reviewed, and approved by the East Carolina University Animal Care and Use Committee.

### Growth measurements

To evaluate growth, the *S. meliloti* strains were first grown on YMB plates for 48 h. The cells were then resuspended in MMNH_4_ to an absorbance of 0.5 at 600 nm, and 2 μl of the cell suspension were added to 200 μl of the test medium. Growth at 30 ^o^C was monitored for 48 h by measuring the absorbance at 600 nm every 30 min using a SPECTRAmax 250 Microplate Spectrophotometer System (Molecular Devices).

Alternatively, cells from 2- to 3-day-old plates were resuspended in Min-salt solution to an absorbance of 0.5 at 600 nm, and the cell suspensions were diluted with Min-salt solution in 1 to 10, 1 to 100, 1 to 1000, 1 to 10,000, and 1 to 100,000 times in a 96-well microplate. Aliquots of these were then transferred using a sterile bolt replicator onto plates containing solid medium. The colony size was scored after 3 to 4 days. The RF requirement of Ba 2308 mutants Bab2_0247 and Bab1_0455 was verified by their ability to grow on SBA plates with the presence and absence of 0.5 mM RF. The strains were grown at 37 ^o^C in the presence of 5% CO_2_ for 48 h.

### Phylogenetic analysis

Visualization of the occurrence and neighborhood of SMc02977 homologs in bacterial genomes was done using STRING, version 11 (ELIXIR) (36). BLASTp searches were carried out on the sequence of SMc02977 against the National Center for Biotechnology Information nonredundant database with the default settings of the BLASTp program. Maximum-likelihood phylogenetic trees based on 1000 bootstrap replicates of the sequence alignment for distinct SMc02977 homologs were constructed using PHYLIP 3.67 (Department of Genome Sciences and the Department of Biology at the University of Washington) (37).

### Expression of endogenous SMc02977 homologs in *S. meliloti*

*B. abortus* bab2_0247 and *L. solanacearum* ckc_00410 ORFs with 500 bp promoter regions were PCR amplified from the genomic DNA. To generate the expression vectors, the resulting PCR fragments were purified using Zymoclean Gel DNA Recovery Kit (ZymoResearch), digested with the HinDIII (for cloning of *B. abortus ribA2*) or BamHI (for cloning of *L. solanacearum ribA2*) restriction enzymes, and subcloned into the HinDIII- or BamHI-digested pCPP30, resulting in plasmids pCPP-Ba-*bab2_0247*-expr and pCPP-Ls-*ckc_00410*-expr, respectively. To overexpress *O. anthropi* American Type Culture Collection 49188 and *M. extorquens* SMc02977 homologs, ORFs of the corresponding genes with 5′ end fused to 176 nucleotides of the SMc02977 promoter region (Genscript) were digested with BamHI restriction enzyme and subcloned into the BamHI-digested pCPP30, resulting in plasmids pCPP-Oa_Oant_3869-expr and pCPP-Me-metdi4691-expr, respectively.

### Bacterial two-hybrid analysis

To detect protein–protein interactions among the RBP enzymes, the bacterial adenylate cyclase two-hybrid system (18) was used as described previously (27). Briefly, the coding regions of *S. meliloti* RF biosynthetic enzymes and SMc02977 and *B. abortus* bab2_0247 were PCR amplified and cloned into the BamHI/KpnI restriction sites of pUT18C and pKT25 plasmids. The constructs were verified by PCR and sequencing. The cya-deficient *E. coli* strain DHM1 was cotransformed with the pUT18C and pKT25 derivatives. Transformants were selected on LB plates supplemented with kanamycin (40 μg/ml), ampicillin (100 μg/ml), and nalidixic acid (15 μg/ml). A positive interaction between two tested proteins produced a functional adenylate cyclase leading to cAMP synthesis and *lacZ* expression. The β-galactosidase activities were determined according to the procedure by Bacterial Adenylate Cyclase Two-Hybrid System kit (Euromedex; catalog no.: EUK001). Measurements of β-galactosidase activity were made in triplicate, and the experiments were repeated at least twice.

### AFRPP synthesis and purification

Briefly, the AFRPP substrate was enzymatically synthesized from GTP using the *Methanocaldococcus jannaschii* GTP cyclohydrolase II (MjGC). To accomplish this, the MjGC coding sequence (encoded by locus MJ0145) was codon optimized for recombinant expression in *E. coli* and ordered synthesized in the peET-30b+ vector from Genscript. The synthesized plasmid was transformed into the BL21(DE3) expression strain of *E. coli* (Stratagene). For heterologous protein expression, *E. coli* was grown in LB (Invitrogen) to an absorbance of 0.6 at 600 nm at 37 °C and then induced with 1 mM IPTG (GoldBio) for 16 h at 37 °C. The induced cells were harvested *via* centrifugation (5500*g* for 15 min at 4 °C) and stored at −80 °C until use.

For lysis and purification, cells were resuspended in binding buffer (20 mM Tris–HCl, pH 7.5, 500 mM NaCl, 20 mM imidazole, pH 7.4, 0.5 mM PMSF, and 10% glycerol) and lysed with 0.1 mm Zirconia/Silica Beads (Biospec Products, Inc) in a mini-beadbeater (Biospec Products). Lysate was clarified at 14,000*g* for 20 min, and the MjGC protein was then His-tag purified on a HiTrap ion affinity chromatography HP column (GE Healthcare) equilibrated in binding buffer on an Akta FPLC (Amersham Biosciences). Purification took place over a gradient of 20 to 500 mM imidazole over 10 CVs at 4 °C. Purified recombinant protein was then desalted into 50 mM TAPS–KOH (pH 8.0) with 15% glycerol, 1 mM KCl, and 6 mM MgCl_2_. The substrate synthesis reaction contained approximately 0.6 mg/ml of purified MjGC protein and 2 mM GTP in 50 mM TAPS–KOH pH 8.0, 10 mM MgCl_2_, 50 mM NH_4_Cl, 10 mM DTT, and was incubated at 60 °C for 6 h. AFRPP was purified away from the MjGC protein and unreacted GTP on a MonoQ 5/50 GL anion exchange column (GE Lifesciences) equilibrated with 25 mM ammonium bicarbonate (pH 7.3) and eluted from the column over a 15 CV linear gradient from 25 mM to 0.5 M ammonium bicarbonate. Fractions containing AFRPP were pooled, spun in the dark under vacuum to dryness, and stored at −80 °C until use.

### Expression of the *S. meliloti* SMc02977 and the *B. abortus* Bab2_0247 in *E. coli*

The ORF was amplified from the *S. meliloti* Rm1021 and *B. abortus* 2308 genomic DNA by PCR using KOD polymerase (Novagen). To generate the expression vector, the resulting PCR fragment was purified using Zymoclean Gel DNA Recovery Kit (ZymoResearch), treated with T4 DNA polymerase (Promega), and then ligated into the pET-30 Ek/LIC vector (Novagen), according to the manufacturer’s protocols. After transformation and recovery, the constructs were verified by DNA sequencing.

### Enzyme purification

LIC_SMc02977 and LIC-Bab2_0247 were transformed into the BL21 strain of *E. coli* (Novagen). The bacterial cultures were grown to an absorbance of 0.6 to 1, and the recombinant protein expression was induced with 1 mM IPTG. The induced cultures were grown for 12 h at 18 °C. Cells were then harvested *via* centrifugation (4000*g* for 10 min at 4 °C), and the recombinant proteins were purified essentially as described previously for MjGC, except that the lysis and column equilibration buffer contained 25 mM Hepes, 300 mM NaCl, 10% glycerol, and 0.25 mM DTT, pH 7.4. The His tags were cleaved off with the recombinant enterokinase (Novagen), and the recombinant proteins containing no tag were desalted into the assay buffer containing 50 mM TES (pH 7.2), 10% glycerol, 0.25 mM DTT, and 1 mM MgCl_2_, loaded on to a MonoQ column equilibrated in the same buffer, and eluted with a linear gradient from 0 to 1 M NaCl. Untagged proteins were desalted a final time into the assay buffer, aliquoted, and stored at −80 °C until use.

### Enzyme assays

Enzymatic hydrolysis of AFRPP to DARoPP was assayed in the assay buffer containing varying substrate concentrations for 8 min at 30 °C. To detect DARoPP, the reaction product was derivatized with 2,3-butanedione to yield a fluorescent product, 6,7-dimethylpterin, which was then quantified using fluorescence HPLC, using the published procedure (4) with the following modifications. The derivatization reaction proceeded with the addition of 34 mM diacetyl in 0.5 M TES (pH 7.2) added at 0.5 v/v to the assays, followed by incubation at 95 °C for 45 min. This was followed by HPLC separation on a Waters Alliance 2695 HPLC system with an Xterra MS C18 column (4.6 × 100 mM, 5 μM) and fluorescence detection (Waters 2475 fluorescence detector) with the excitation wavelength of 330 nm and emission wavelength of 435 nm. The HPLC separation was conducted isocratically in a mobile phase consisting of 3% (v/v) methanol and 97% 27 mM phosphoric acid. A standard curve was constructed from known concentrations of 6-hydroxy-2,4,5-triamino-pyrimidine sulfate (Aldrich) following the published procedure (4). To detect formate, a published procedure (38) was used with the following modifications: To 25 μl of the assay mixture was added 25 μl of 20 mM tetrabutylammonium bromide in acetonitrile, 25 μl of 2.5% triethanolamine in acetonitrile, and 5 μl of 9-chloromethyl anthracene in acetonitrile. Next, the total volume was brought to 250 μl with acetonitrile, and the mixture was incubated at 75 °C for 50 min. The HPLC separation was conducted on the Xterra MS C_18_ column (3.5 μm, 4.6 × 100 mm) isocratically with a 1.0 ml/min flow rate, with a mobile phase consisting of 64% acetonitrile and 36% water. Quantification was based on a comparison with the standards obtained by derivatizing pure formic acid (Sigma). Data are average of three assays done in duplicate.

### DARoPP extraction and quantification

The *S. meliloti* pellets were resuspended in a solution of 9:10 methanol: methylene chloride (v:v) at 5 ml per gram of bacterial pellet. The samples were then vortexed at maximum speed for 1 min before addition of 0.1 M (pH 6.0) ammonium acetate at 2.5 ml per gram of bacterial pellet. After vortexing for an additional minute, centrifugation at 14,000*g* for 15 min was used to separate the organic and aqueous layers. The aqueous layer was removed and evaporated to dryness in a rotary evaporator. The residue was then resuspended directly in 0.25 M TES (pH 7.2) containing 34 mM diacetyl, followed by incubation at 95 °C for 45 min. Quantitative HPLC analysis to detect derivatized DARoPP was conducted as described previously for the enzyme assays. Data are average of four experiments.

## Data availability

All data are contained within the article or supporting information.

## Supporting information

This article contains supporting information (9, 17, 33, 39, 40).

## Conflict of interest

The authors declare that they have no conflicts of interest with the contents of this article.
