# Supplementary material for: A novel formamidase is required for riboflavin biosynthesis in invasive bacteria
Source: J Biol Chem. 2022 Aug 13;298(9):102377. doi: 10.1016/j.jbc.2022.102377 (PMC9478397; doi:10.1016/j.jbc.2022.102377)
Supplement: Table_S1 [file mmc1.pdf]

Table S1. *S. meliloti* and *B. abortus rib* mutants

Strain name	Genotype	Requirement of RF for growth
Rm1021	Wild type *S. meliloti*	No
Rm1021Δ*ribBA*-RFA-12	SMc02977-3098741*::Tn5-mini mutant of Rm1021Δ*ribBA*	Yes
Rm1021Δ*ribBA*-RFA-Σ	SMc02977-3098830::Tn5-mini mutant of Rm1021Δ*ribBA*	Yes
Td-Rm1021-RFA-12	Transductant of SMc02977-3098741::Tn5 into Rm1021	Yes
Td-Rm1021-RFA-Σ	Transductant of SMc02977-3098830::Tn5 into Rm1021	Yes
Rm1021ΔSMc02977	SMc02977 deletion mutant of Rm1021	Yes
Rm1021Δ*ribBA*ΔSMc02977	SMc02977 deletion mutant of Rm1021Δ*ribBA*	Yes
Rm1021ΔSMc02977(pCPP-Ba-*bab2_0247*-expr)	Rm1021ΔSMc02977 overexpressing SMc02977 homolog from *B. abortus*	No
Rm1021ΔSMc02977(pCPP-Ls-*ckc_00410*-expr)	Rm1021ΔSMc02977 overexpressing SMc02977 homolog from *L. solanacearum*	No
Rm1021ΔSMc02977(pCPP-Me-*metdi4691*-expr)	Rm1021ΔSMc02977 overexpressing SMc02977 homolog from *M. extorquens*	Yes
Rm1021ΔSMc02977(pCPP-Oa_*Oant_3869*-expr)	Rm1021ΔSMc02977 overexpressing SMc02977 homolog from *O. anthropi*	Yes
Rm1021ΔSMc02976	SMc02976 deletion mutant of Rm1021	No
Rm1021ΔSMc02978	SMc02978 deletion mutant of Rm1021	No
JB32	bab2_0247 deletion mutant of Ba 2308	Yes
JB24	bab1_0455 deletion mutant of Ba 2308	No

*The numbers indicate the position of the transition from the Tn5 shoulder to the *S. meliloti* Rm1021 sequence
