# Supplementary material for: A novel formamidase is required for riboflavin biosynthesis in invasive bacteria
Source: J Biol Chem. 2022 Aug 13;298(9):102377. doi: 10.1016/j.jbc.2022.102377 (PMC9478397; doi:10.1016/j.jbc.2022.102377)
Supplement: Table S2 [file mmc2.pdf]

Table S2. Bacterial strains and plasmids.

Strains	Genotype or characteristics	Source
Sinorhiozbium meliloti		
Rm1021	Wild type	(Galibert, Finan et al. 2001)
Rm1021Δ*ribBA*	*ribBA* deletion mutant of Rm1021	(Yurgel, Rice et al. 2014)
Rm1021Δ*ribBA*-RFA-12	SMc02977-3098741*::Tn5-mini mutant of Rm1021Δ*ribBA*, RF auxotroph	This work
Rm1021Δ*ribBA*-RFA-Σ	SMc02977-3098830::Tn5-mini mutant of Rm1021Δ*ribBA*, RF auxotroph	This work
Td-Rm1021-RFA-12	Transductant of SMc02977-3098741::Tn5 into Rm1021, RF auxotroph	This work
Td-Rm1021-RFA-Σ	Transductant of SMc02977-3098830::Tn5 into Rm1021, RF auxotroph	This work
Rm1021ΔSMc02977	SMc02977 deletion mutant of Rm1021, RF auxotroph	This work
Rm1021ΔSMc02976	SMc02976 deletion mutant of Rm1021	This work
Rm1021ΔSMc02978	SMc02978 deletion mutant of Rm1021	This work
Brucella abortus		
B. abortus 2308	Wild type	
JB32	Bab2_0247(Sm-BrbF homolog) deletion in Ba2308, RF auxotroph	This work
JB24	Bab1_0455(RibBA) deletion in Ba2308	This work
E. coli		
BL21	*fhuA2 [lon] ompT gal [dcm] ΔhsdS*	Invitrogen
S17-1	*pro hsdR recA* [RP4-2(Tc::Mu) (Km::Tn7)]	(Simon, Quandt et al. 1989)
Plasmids		
pJG110	Mini-Tn*5*/Tn5-110 transposon delivery vector (Km/NmR, ApR)	(Griffitts and Long 2008)
pCPP30	IncP LacZ; Tc	(Huang, He et al. 1992)

pCPP-Ba-*bab2_0247*-expr	pCPP30[*B. abortus 2308* bab2_0247 with 500 bp promoter region]	This work
pCPP-Ls-*ckc_00410*-expr	pCPP30[*L. solanacearum* ckc_00410 with 500 bp promoter region]	This work
pCPP-Me-*metdi4691*-expr	pCPP30[*M. extorquens* METDI4691 with 500 bp promoter region of SMc02976]	This work
pCPP-Oa_*Oant_3869*-expr	pCPP30[*O. anthropi* Oant_3869 with 500 bp promoter region of SMc02976]	This work
pK19*mobsacB*	pK19*mob* derivative *sacB*	(Schafer, Tauch et al. 1994)
pNPTS193	Cloning vector; contains sacB gene	(Spratt, Hedge et al. 1986)
pNPTSΔ0455	pNPTS193 [1000bp flanking *B. abortus* bab1_0455]	This work
pNTPSΔ0247	pNPTS938 [1000bp flanking *B. abortus* bab2_0247]	This work
pK19mobsacB-ΔSMc02977	pK19*mobsacB* [500/600bp flanking *S. meliloti* Smc02977]	This work
pK19mobsacB-ΔSMc02976	pK19*mobsacB* [500/600bp flanking *S. meliloti* Smc02976]	This work
pK19mobsacB-ΔSMc02978	pK19*mobsacB* [500/600bp flanking *S. meliloti* Smc02978]	This work
LIC_SMc02977	pET-30 Ek/LIC, *S. meliloti* SMc02977 expression	This work

*The numbers indicate the position of the transition from the Tn5 shoulder to the *S. meliloti* Rm1021 sequence

Galibert, F., T. M. Finan, S. R. Long, A. Puhler, P. Abola, F. Ampe, F. Barloy-Hubler, M. J. Barnett, A. Becker, P. Boistard, G. Bothe, M. Boutry, L. Bowser, J. Buhrmester, E. Cadieu, D. Capela, P. Chain, A. Cowie, R. W. Davis, S. Dreano, N. A. Federspiel, R. F. Fisher, S. Gloux, T. Godrie, A. Goffeau, B. Golding, J. Gouzy, M. Gurjal, I. Hernandez-Lucas, A. Hong, L. Huizar, R. W. Hyman, T. Jones, D. Kahn, M. L. Kahn, S. Kalman, D. H. Keating, E. Kiss, C. Komp, V. Lelaure, D. Masuy, C. Palm, M. C. Peck, T. M. Pohl, D. Portetelle, B. Purnelle, U. Ramsperger, R. Surzycki, P. Thebault, M. Vandenbol, F. J. Vorholter, S. Weidner, D. H. Wells, K. Wong, K. C. Yeh and J. Batut (2001). "The composite genome of the legume symbiont Sinorhizobium meliloti." Science **293**(5530): 668-672.

Griffitts, J. S. and S. R. Long (2008). "A symbiotic mutant of Sinorhizobium meliloti reveals a novel genetic pathway involving succinoglycan biosynthetic functions." Mol Microbiol **67**(6): 1292-1306.

Huang, H. C., S. Y. He, D. W. Bauer and A. Collmer (1992). "The Pseudomonas syringae pv. syringae 61 hrpH product, an envelope protein required for elicitation of the hypersensitive response in plants." J Bacteriol **174**(21): 6878-6885.

Schafer, A., A. Tauch, W. Jager, J. Kalinowski, G. Thierbach and A. Puhler (1994). "Small mobilizable multi-purpose cloning vectors derived from the Escherichia coli plasmids pK18 and pK19: selection of defined deletions in the chromosome of Corynebacterium glutamicum." Gene **145**(1): 69-73.

Simon, R., J. Quandt and W. Klipp (1989). "New derivatives of transposon Tn5 suitable for mobilization of replicons, generation of operon fusions and induction of genes in gram-negative bacteria." Gene **80**(1): 161-169.

Spratt, B. G., P. J. Hedge, S. te Heesen, A. Edelman and J. K. Broome-Smith (1986). "Kanamycin-resistant vectors that are analogues of plasmids pUC8, pUC9, pEMBL8 and pEMBL9." Gene **41**(2-3): 337-342.

Yurgel, S. N., J. Rice, E. Domreis, J. Lynch, N. Sa, Z. Qamar, S. Rajamani, M. Gao, S. Roje and W. D. Bauer (2014). "Sinorhizobium meliloti flavin secretion and bacteria-host interaction: role of the bifunctional RibBA protein." Mol Plant Microbe Interact **27**(5): 437-445.
