# Supplementary material for: A novel formamidase is required for riboflavin biosynthesis in invasive bacteria
Source: J Biol Chem. 2022 Aug 13;298(9):102377. doi: 10.1016/j.jbc.2022.102377 (PMC9478397; doi:10.1016/j.jbc.2022.102377)
Supplement: Table S3 [file mmc3.pdf]

Table S3. Primers used in this study

The restriction sites are underlined.

Name	Sequence 5` -> 3`	Experiment
SMc02977_del_F-1	CATGGAATTCAGCTCTGTCAGATAGACCTTGGT	SMc02977 in-frame deletion, *S. meliloti*
SMc02977_del_R_1	CATGGGATCCTTCTACTCGGCCCTCGGCGTCGT	
SMc02977_del_F_2	CATGGGATCCATGCCGGCGAGCTCGACTGA	
SMc02977_del_R_2	CATGGAATTCCATCAGTCCTTCGACGACAC	
SMc02977_ver_F	CTTCCGCGATGATCTGCAGA	
SMc02977_ver_R	GCGTCGAAGCGTCCGGGACG	
SMc02976_del_F-1	CATGGAATTCGTTCGACAGGCAGGAAGGTCACG	SMc02976 In-frame deletion, *S. meliloti*
SMc02976_del_R_1	CATGGGATCCTGGACCTCCCGCCGAATCCCCTACACT	
SMc02976_del_F_2	CATGGGATCCTTGAGCCGGGGTTTGAGAACCG	
SMc02976_del_R_2	CATGGAATTCACCGAGCTGCAGCAGCGTCTCGAT	
SMc02976_ver_F	ACATCCATGTGTTCGATCGAA	
SMc02976_ver_R	GATAGCGGGCGACATGATCAC	
SMc02978_del_F-1	CATGGAATTCGCGCTTTCCTTCAAGCATCTGC	SMc02978 in-frame deletion, *S. meliloti*
SMc02978_del_R_1	CATGGGATCCGGGAACCTCGAGGGTTGGCGAT	
SMc02978_del_F_2	CATGGGATCCGGCATGATCCCGGAAGATGGCG	
SMc02978_del_R_2	CATGGAATTCGTAGGCGTAAGCGACCGCACCTT	
SMc02978_ver_F	CATCCGGACAAGGTGGATAT	
SMc02978_ver_R	GGACATGCGTCGTCTTGCCGT	
BAB2_0247 Up Fwd	TGATGGTGATGGTCATGCCC	BAB2_0247 in-frame deletion, *B. abortusi*
BAB2_0247 Up Rev	AAAACATGACCGATCTGCGCGCATGAACATGATCCG GCTG	
BAB2_0247 Dwn Fwd	CAGCCGGATCATGTTCATGCGCGCAGATCGGTCATGT TTT	
BAB2_0247 Dwn Rev	AACGCGAGGTACGAAGGCTC	
BAB1_0455 Up Fwd	TGCTTTCCGGCGTGCTTCTG	BAB1_0455 in-frame deletion, *B. abortusi*
BAB1_0455 Up Rev	TTGGGAGACATGCATTAGATCGCATCAACGACCTGTT TCTGA	
BAB1_0455 Dwn Fwd	TCAGAAACAGGTCGTTGATGCGATCTAATGCATGTCT CCCAA	
BAB1_0455 Dwn Rev	GACTCATCGAGATTGATCTC	
Ls_ckc_00410_expr_F	ATGCGGATCCCGATAGCGGCTGCCATTTGT	Functional complementation of SMc02977 in pCPP30, *S. meliloti*
Ls_ckc_00410_expr_R	ATGCGGATCCAGCGCCTATCGGAACTATTC	
BA_BAB2_0247_expr_F	ATGCAAGCTTGAAGCGCGCTTGACCACAAATCC	
BA_BAB2_0247_expr_R	ATGCAAGCTTTATCACACGCAGCCGGATCATGT	
Me_METDI4691_F	CATGGAATTCGGATCCGACGATCTCGTCCTTCTTCT	
Me_ METDI4691_R	CATGGAATTCGGATCCTCAGGCTCCTGGCATGGGGA	

Oa_Oant_3869_F	ATGCGGATCCGGCGACGATCTCGTCCTTCT	
Oa_Oant_3869_R	ATGCGGATCCTTACCAGGCTGGTTTGTTGT	
PET30LIC_cahA_F	GACGACGACAAGATGTCGTTGCCGAAGCCCCGATGG	Cloning in pET30 for protein expression, *S. meliloti* ArfB
PET30LIC_cahA_R	GAGGAGAAGCCCGGTCAGTCGAGCTCGCCGGCATCGA	
PET30LIC_BAB2_0247_F	GACGACGACAAGATGACCGATCTGCGATCAG	Cloning in pET30 for protein expression, *B. abortus*
PET30LIC_BAB2_0247_R	GAGGAGAAGCCCGGTCATGCCATCACTTTCGGGTTG	
Sm_ SMc02977_BTH_F	ATGCGGATCCCATGTCGTTGCCGAAGCCCCGAT	Bacterial two hybrid experiments, *S meliloti*
Sm_ SMc02977_BTH_R	ATGCGGTACCCGGTCGAGCTCGCCGGCATCGA	
Ba_ BAB2_0247_BTH_F	ATGCGGATCCCATGACCGATCTGCGCGATCA	Bacterial two hybrid experiments, *B. abortus*
Ba_ BAB2_0247_BTH_R	ATGCGGTACCCGTGCCATCACTTTCGGGTTGA	
