# Supplementary material for: A novel formamidase is required for riboflavin biosynthesis in invasive bacteria
Source: J Biol Chem. 2022 Aug 13;298(9):102377. doi: 10.1016/j.jbc.2022.102377 (PMC9478397; doi:10.1016/j.jbc.2022.102377)
Supplement: Fig_S1 [file mmc4.pdf]

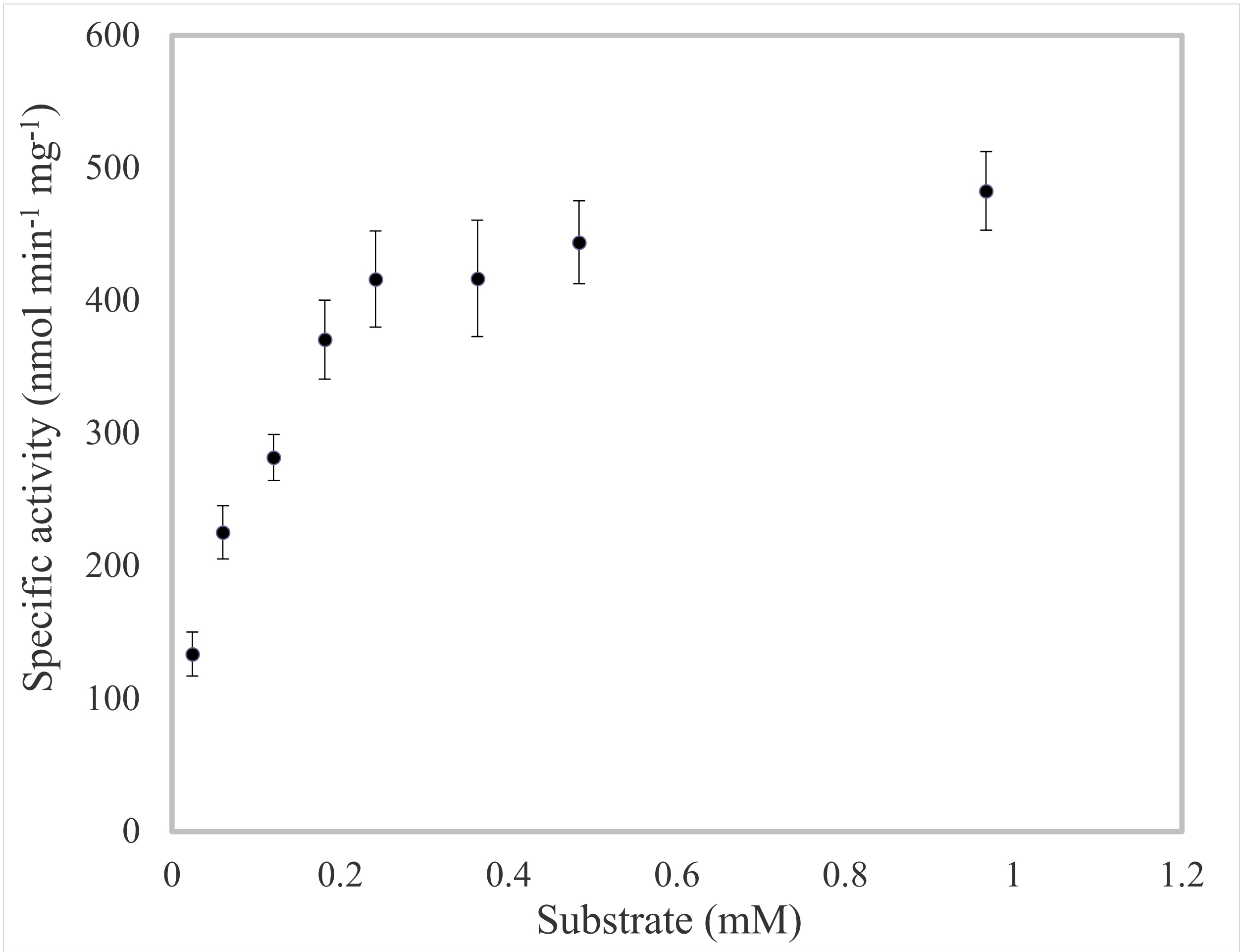

Fig. S1. Dependence of specific activity of Sm-BrbF on AFRPP concentration. Measurements were made at pH 7.5 with various AFRPP concentrations. Curves are nonlinear best fits to the Michaelis–Menten model (SigmaPlot 9.0.1). Data are the average ± standard error (SE) of five triplicate determinations.
