# Supplementary material for: A novel formamidase is required for riboflavin biosynthesis in invasive bacteria
Source: J Biol Chem. 2022 Aug 13;298(9):102377. doi: 10.1016/j.jbc.2022.102377 (PMC9478397; doi:10.1016/j.jbc.2022.102377)
Supplement: Fig_S2 [file mmc5.pdf]

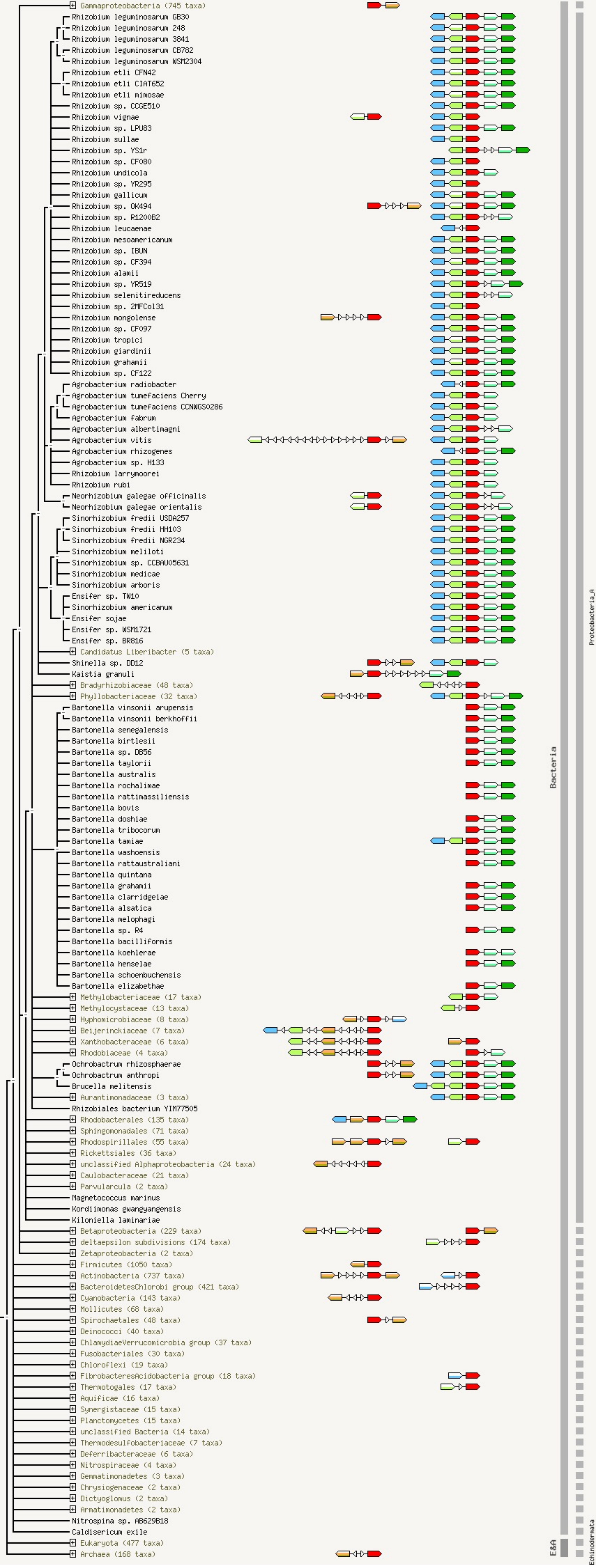

Fig. S2. Occurrence and neighborhood of SMc02977 homologs in bacterial genomes. STRING v11 (Szklarcyk et al., 2019) software was used to generate the visualization. Red boxes indicate SMc02977 homologs; Blue boxes indicate SMc02976 homologs; and White/turquois boxes indicate SMc02978 homologs.
