# Supplementary figures and images for: A novel formamidase is required for riboflavin biosynthesis in invasive bacteria
Source: J Biol Chem. 2022 Aug 13;298(9):102377. doi: 10.1016/j.jbc.2022.102377 (PMC9478397; doi:10.1016/j.jbc.2022.102377)

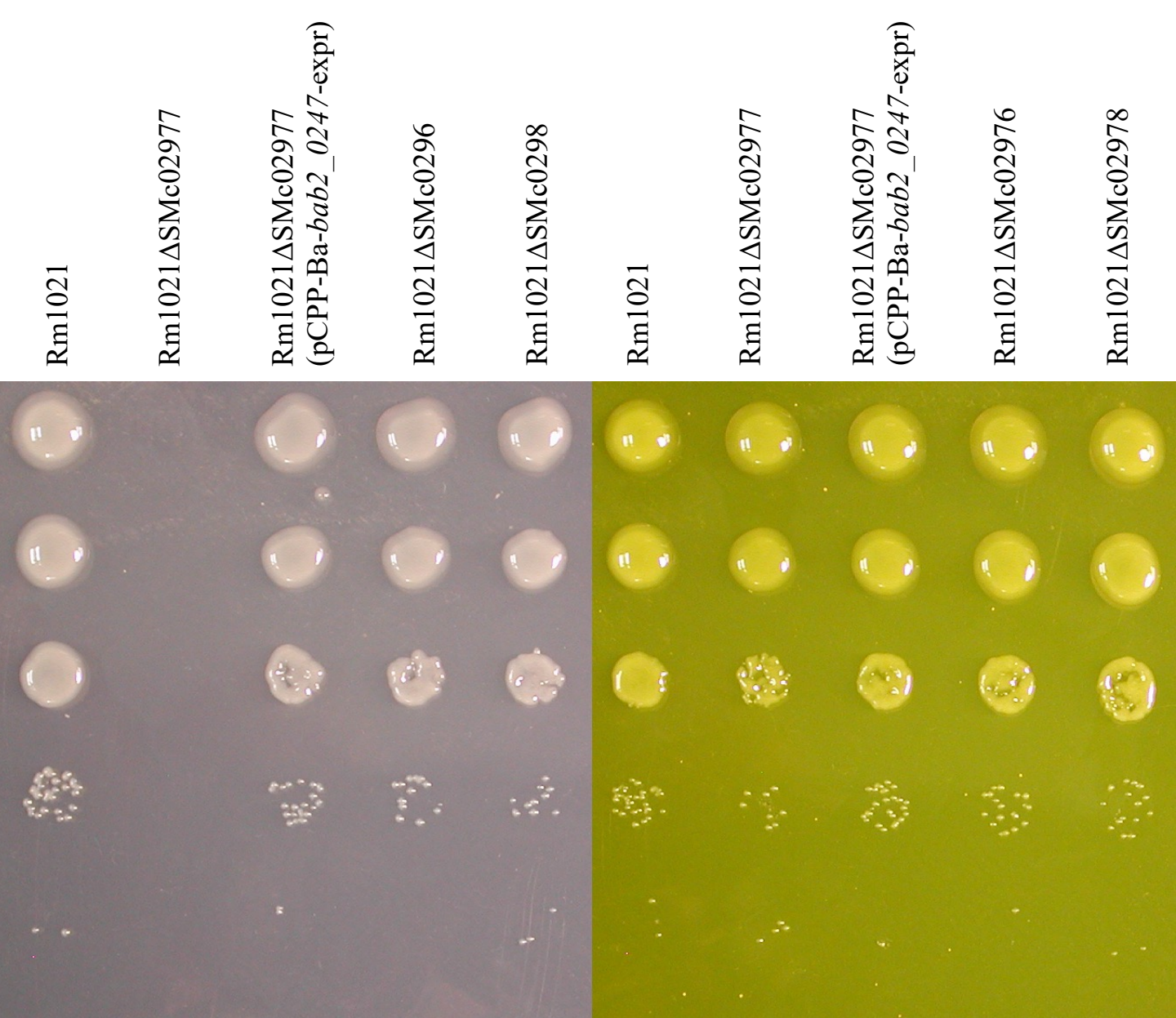

Fig. S3. Growth properties of Rm1021 strain.  
Left panel – LB; Right panel – LB supplemented with 250  $\mu$ M RF

Supplement: Fig_S3 [file mmc6.pdf]
