# Supplementary material for: A novel formamidase is required for riboflavin biosynthesis in invasive bacteria
Source: J Biol Chem. 2022 Aug 13;298(9):102377. doi: 10.1016/j.jbc.2022.102377 (PMC9478397; doi:10.1016/j.jbc.2022.102377)

Fig. S4. Reactions catalyzed by GCHII (right panel, KEGG Reaction R00425) and creatininase (left panel, KEGG Reaction R01884).

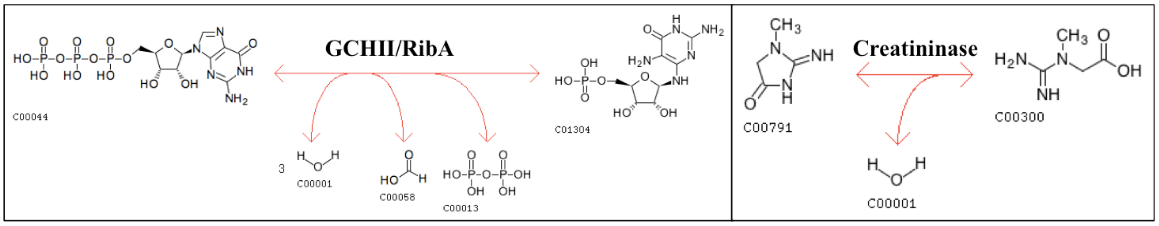

Supplement: Fig_S4 [file mmc7.pdf]
