# Supplementary material for: A novel formamidase is required for riboflavin biosynthesis in invasive bacteria
Source: J Biol Chem. 2022 Aug 13;298(9):102377. doi: 10.1016/j.jbc.2022.102377 (PMC9478397; doi:10.1016/j.jbc.2022.102377)
Supplement: Fig_S5 [file mmc8.pdf]

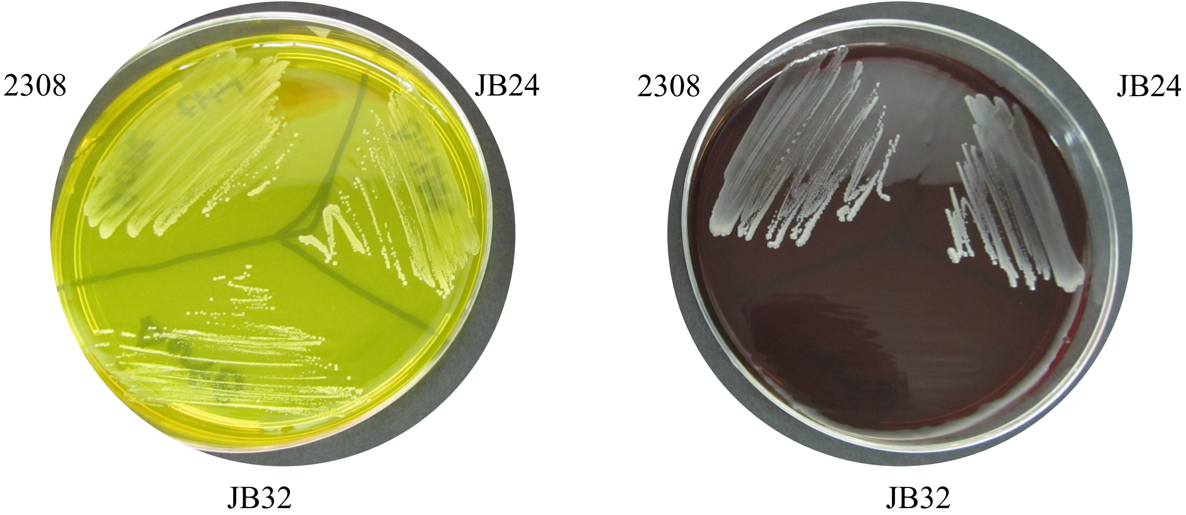

Fig. S5. Growth properties of *B. abortus* strains.
Left panel – SA media supplemented with 250 μM RF; Right panel – SBA media
