# Supplementary material for: A novel formamidase is required for riboflavin biosynthesis in invasive bacteria
Source: J Biol Chem. 2022 Aug 13;298(9):102377. doi: 10.1016/j.jbc.2022.102377 (PMC9478397; doi:10.1016/j.jbc.2022.102377)
Supplement: Fig_S6 [file mmc9.pdf]

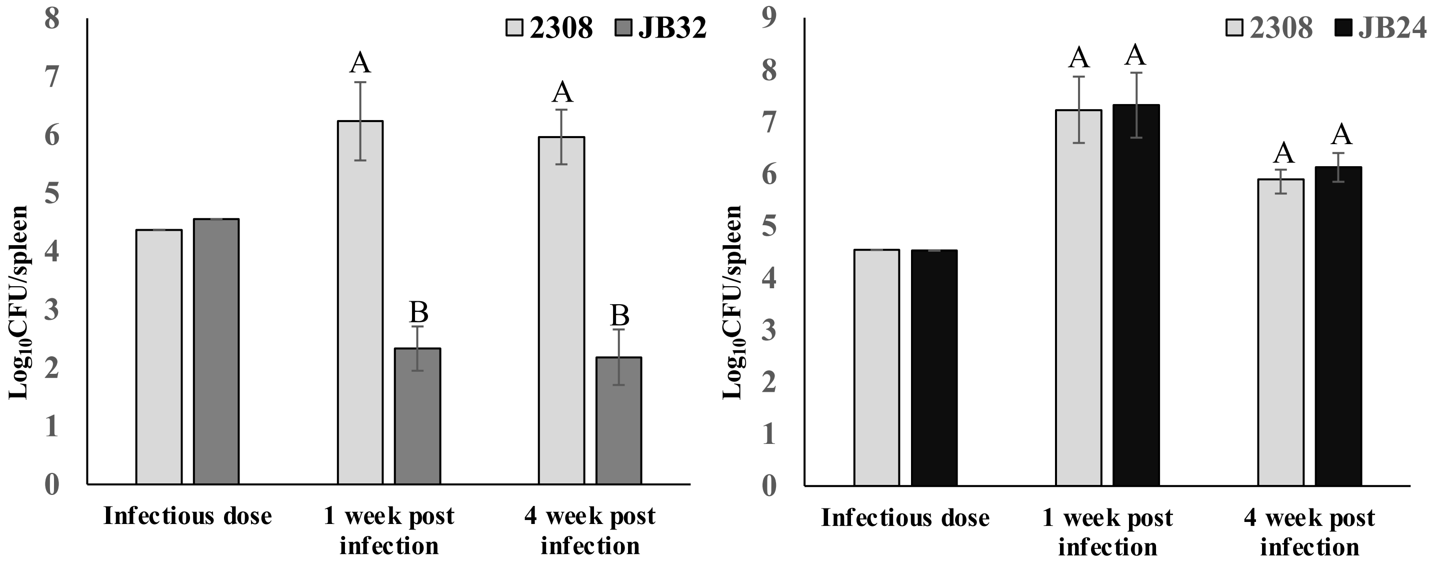

Fig. S6. The *B. abortus arfB* mutant displays attenuation in mice. Spleen colonization profiles of *Brucella abortus* 2308, the *arfB* mutant (JB32, left panel), and *ribBA* mutant (JB24, right panel) in C57BL/6$^{Nramp1+/+}$ mice are shown. Ten mice were individually infected with each bacterial strain via the intraperitoneal route, and five mice from each experimental group were evaluated at 1 weeks and 4 weeks post-infection. The values within each time point group labelled with the different letters are significantly different based on Student's T-Test, p-value<0.001. Data are the average ± standard deviation (SD). Three biological replicates were used for each measurement.
